# Vegetation Health Indicators of Groundwater Discharge: Integration of Sentinel-2 Remote Sensing and Meteorological Time Series in the Northern Apennines (Italy)

**DOI:** 10.3390/s26051464

**Published:** 2026-02-26

**Authors:** Murad Abuzarov, Stefano Segadelli, Duccio Rocchini, Marco Cantonati, Alessandro Gargini

**Affiliations:** 1 Department of Biological, Geological and Environmental Sciences, Alma Mater Studiorum University of Bologna, 40126 Bologna, Italy; murad.abuzarov@studio.unibo.it (M.A.); duccio.rocchini@unibo.it (D.R.); alessandro.gargini@unibo.it (A.G.); 2 Geology, Soil and Seismic Area, Emilia-Romagna Region, 40127 Bologna, Italy; stefano.segadelli@regione.emilia-romagna.it; 3 Department of Spatial Sciences, Czech University of Life Sciences, 165 00 Prague, Czech Republic

**Keywords:** remote sensing, NDVI, Spring, groundwater, Ecohydrogeology, drought, climate change

## Abstract

**Highlights:**

**What are the main findings?**

- The NDVI-derived vegetation anomalies indicate zones influenced by groundwater discharge within forested regions during droughts;
- Groundwater discharge areas enhance vegetation resilience under severe drought conditions in a Mediterranean mountain setting.

**What are the implications of the main findings?**

- Remote sensing–based NDVI analysis can support reconnaissance and inventory of potential groundwater discharge zones in remote and rugged landscapes;
- Springs, including tapped springs used for human supply, represent key groundwater-dependent ecosystems in forested regions and require protection.

**Abstract:**

This study evaluates the capability of multi-temporal vegetation indices derived from Sentinel-2 imagery to indicate groundwater discharge in a forested mountainous sector of the Northern Apennines (Italy). The NDVI was computed from Level-2A surface reflectance data (10 m resolution) and analyzed over five growing seasons (2017–2021), encompassing recurrent summer droughts. Aridity conditions were quantified using the Standardized Precipitation–Evapotranspiration Index (SPEI) derived from long-term meteorological records. The methodological framework integrates cloud-masked satellite observations, drought characterization, and spatial statistical comparison between known spring discharge zones and randomly distributed forested control points. NDVI values extracted within 100 m radius buffers, centered on spring outlets, were systematically compared with those from control areas located outside the shallow-water-table influence zone. During periods of negative SPEI (moderate-to-severe drought), spring-centered buffers consistently exhibited higher NDVI values than control sites, with the NDVI contrast increasing under severe arid conditions. This pattern indicates enhanced vegetation resilience supported by shallow groundwater availability. The results demonstrate that vegetation health anomalies, when constrained by homogeneous land cover and a consistent hydrogeological setting, can serve as indicators of the groundwater discharge likelihood. The proposed workflow provides a reproducible and cost-effective tool to support hydrogeological reconnaissance and spring inventorying in rugged mountainous environments where field-based surveys are logistically demanding.

## 1. Introduction

Springs, as highlighted by [1], are key ecotones at the transition between groundwater and surface water, providing a wealth of ecosystem services thanks to the high-quality water they discharge, which also generates environmental flows crucial for maintaining streams and surface aquatic ecosystems. Moreover, in many regions of the world, particularly in arid and sub-tropical countries, springs provide high-value water for human consumption. Therefore, in the face of climate change and recurrent extreme hydrological events, like droughts, the role of springs is becoming increasingly strategic [2,3,4,5,6]. Whereas water shortages particularly affect surface water systems or shallower aquifers, some springs could prove highly resilient to the effects of climate change, being connected to significantly larger groundwater reservoirs and flow systems [7].

High greenhouse-gas emission scenarios are likely to intensify and further increase the occurrence of droughts [8,9]. Under the IPCC (Intergovernmental Panel on Climate Change)-defined RCP 4.5 (Representative Concentration Pathway) scenario—which represents a moderately optimistic assessment—projections indicate a significant increase in drought frequency by the end of the century in southern and western Europe compared to recent times [10,11]. Ref. [12] characterizes drought, a general term, as an extreme climate event involving prolonged below-average rainfall that disrupts the hydrological balance, leading to a substantial reduction in moisture, relative to a standard baseline, and diminished water storage in reservoirs [13]. Moreover, ref. [14] indicates that, with global warming, there is a consistent upward trend in Potential Evapotranspiration (PET) across all major regions, which, without a corresponding rise in precipitation, is likely to exacerbate drought conditions due to increased surface drying. From a quantitative hydrological standpoint, the Standardized Precipitation–Evapotranspiration Index (SPEI) is considered a robust indicator of drought conditions [15]. The SPEI leverages the difference between available water, expressed by Precipitation (P), and Potential Evapotranspiration (PET)—the latter estimated via Hargreaves’ formula [16]—in order to assess aridity conditions for a specific location over varying timescales, from 1 to 48 months. The SPEI range, spanning from −2 to +2, distinctly separates dry and wet conditions, respectively [17].

Drought is an environmental hazard that has an immense negative impact on vegetation [18]. Many ecosystems, particularly forests, have evolved to cope with regular water shortages through a range of adaptations [19], but extreme or prolonged droughts can push these systems beyond their capacity to adjust, leading to significant hydrological and ecological changes [20,21]. For this reason, the water-related ecosystem services provided by rivers, lakes, springs, and wetlands are even more vital for the vegetation, particularly during drought periods [22,23,24]. In mountainous settings, the groundwater discharge points—where the water table approaches from below to intersect the ground surface—are spots where the vegetation is less stressed and capable of gaining more water for transpiration, creating distinctive mesic microhabitats that provide water resources and contrast the effects of drought [25,26]. When groundwater is accessible to tree roots, with a depth to water table lower than 5 m below the ground surface (as expected in groundwater discharge outlets), this is enough to relatively mitigate water shortage effects in forest ecosystems [27,28,29,30,31,32,33,34]. Thus, during droughts, the greenness of vegetation in these spots should stand out compared to nearby areas. Indeed, phreatophytic plant associations are an evident example of a subaerial groundwater-dependent ecosystem totally supported by a low depth-to-water-table setting.

Remote-sensing-derived vegetation indices play a crucial role in precisely evaluating plant growth conditions and vegetation stress, particularly during extreme weather phenomena like droughts [35]. The light spectrum that plants reflect ranges from visible to near-infrared. In the case of plant leaves, chlorophyll captures visible light (ranging from 0.4 to 0.7 μm) for the process of photosynthesis, with higher absorption indicating greater productivity, whilst the near-infrared light (spanning 0.7 to 1.1 μm) is reflected [36]. The Normalized Difference Vegetation Index (NDVI) serves as a key indicator in satellite-based vegetation surveys of plant health and productivity [37]. It is expressed as NDVI = (ρNIR − ρRED) / (ρNIR + ρRED), where ρRED and ρNIR stand for the acquired spectral reflectance measurements in the red (visible) and near-infrared regions, respectively. The spectral reflectance values are themselves ratios of the reflected radiation to the incoming radiation in each spectral band, with values between 0 and 1. The NDVI yields values within a range from −1 to +1. Scores nearing zero indicate a lack of green vegetation, whereas values closer to +1 (around 0.8–0.9) denote the maximum density of greenery. The NDVI effectively acts as a proxy for climatic and hydrological conditions affecting vegetation functionality, and so it reflects the stress from drought conditions [38,39]. The use of the NDVI to remotely monitor plants’ phenology near to discharge points has been considered by [40]. The potential of spring areas to behave as climatic refugia in semi-arid environments has been verified in Idaho (USA) by the use of an NDVI comparison with non-spring areas [26], and other authors have identified a relationship between the NDVI and the areal density of springs [41]. The NDVI indicator appears to be particularly valuable when assessed relative to natural forested areas rather than human-impacted ones, where the effects of croplands or urbanization may result in competition between species and exacerbate the effects of droughts, resulting in a more pronounced decrease in vegetation health and continuity [42].

If the relevance of groundwater in addressing adaptation strategies to climate extremes is becoming paramount, the quantitative assessment of below-ground water flows is still very poor, and more efforts are needed to map springs in order to foster their proper management. The current methods for identifying the location of springs in wild and mountainous settings are expensive and challenging. Considering the strict relationship between groundwater discharge outlet points and vegetation productivity, remote-sensing-derived indices of vegetation stress could be evaluated as a proxy to locate groundwater discharge and also for uptake springs.

This study presents a remote-sensing-based approach, integrating the Normalized Difference Vegetation Index (NDVI)—derived from Sentinel-2 imagery in the red and near-infrared bands—with the spatial distribution of groundwater discharge outlets (springs) in forested environments. The objective is to evaluate whether NDVI anomalies, enhanced by the presence of a shallow water table during drought conditions, can serve as indicators of groundwater discharge influence. The central hypothesis is that continuous forest cover located near discharge outlets experiences reduced water stress compared to surrounding areas. Drought severity is characterized using the Standardized Precipitation–Evapotranspiration Index (SPEI). The selected study area in the Northern Apennines (Italy) is particularly suitable because it is periodically affected by intense and prolonged droughts, with documented impacts on hydrological processes and forest ecosystems [7,43,44], and because a detailed census and monitoring dataset of the springs is available. In contrast to previous studies that applied NDVI to detect natural free-flowing discharge points in predominantly arid environments [26], this work focuses on tapped springs (spring-boxes) and evaluates the approach in a more mesic climatic setting (annual precipitation of 600–1000 mm) that is nonetheless subject to recurrent severe droughts.

## 2. Materials and Methods

**Figure 1:**
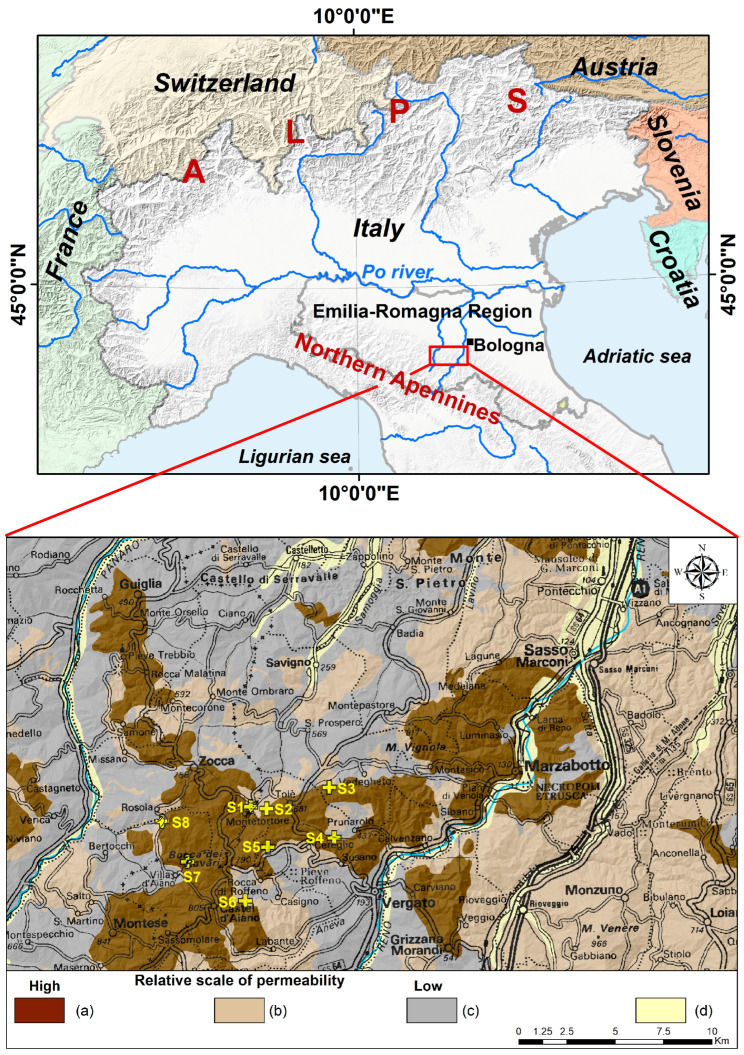
(**top**) Location of the study area (red rectangle) in the wider geographical region; (**bottom**) detail of the study area with geological mapping of aquifer units (colors a and b) and aquitard/aquiclude units (low permeability, color c). River valleys’ unconsolidated deposits (d). Investigated springs are marked with yellow crosses. Pantano Formation aquifer is in brown.

The study was conducted in a sector of Northern Apennines in the Emilia-Romagna Region (Italy; Figure 1). The Apennines constitute a Neogene fold-thrust belt, formed during the Alpine orogenesis due to the continental collision between the Adria microplate (a fragment of the African plate) and the Eurasian plate [45,46]. The main outcropping lithologies of the chain are represented by alternating sandstones and mudstones (turbidites) with a bulk medium–low permeability. The main aquifers, i.e., geological units with relatively higher permeability, are scattered around and represented by arenites and ophiolites (magmatic rocks) [4].

In the investigated sector, located between the Reno and Panaro river valleys in a medium-elevation mountainous setting, groundwater flow systems discharge from the Pantano Formation (PAT) (Figure 1), one of the primary regional aquifers, consisting of a fractured medium-to-fine-grained calcarenite (calcite-rich sandstone) formed in a shallow marine setting during the Lower to Middle Miocene [4,47]. The aquifer is subjected to karst enlargement of discontinuities by corrosion, with a consequent increase in permeability.

The climate zone of the area, according to the Köppen–Geiger classification [48], is categorized as C_fb_, warm temperate, distinguished by mild winters, with average daily lows (T_min_) between −2 °C and 4 °C, and cool summers, with average peak temperatures (T_max_) between 26 °C and 28 °C. Typical of the Mediterranean-type facies of the climate is the markedly dry season between June and October (occasionally interrupted by thunderstorms, generally insufficient to induce groundwater recharge). Following local climatic patterns, spring discharge typically peaks in spring, following the November to April wet season, and is lowest during the dry July–September period [49,50].

A total of eight springs were selected for the investigation, from a total of 41 identified from a preceding survey, and these were all subjected to hydrologic monitoring of the total flow rate discharge along the hydrological year 2020–2021 (from October 2020 to September 2021), notably during a significantly arid year, characterized by a marked reduction in seasonal precipitation and a rise in average annual temperatures [4]. The springs are located between 543 and 714 m a.s.l., along the geological contact between the Pantano Formation and the low-permeability units around (Figure 1), and are partially captured by spring-boxes for the local aqueducts’ drinking water supply. The reason for their selection is that they are the most completely encircled, in their discharge hot-spot, by continuous vegetation cover, represented locally by a predominant oak and hornbeam forest with scattered chestnut groves and with minimal interference of human-induced land-use changes, such as agriculture, urbanization, and infrastructure development.

**Figure 2:**
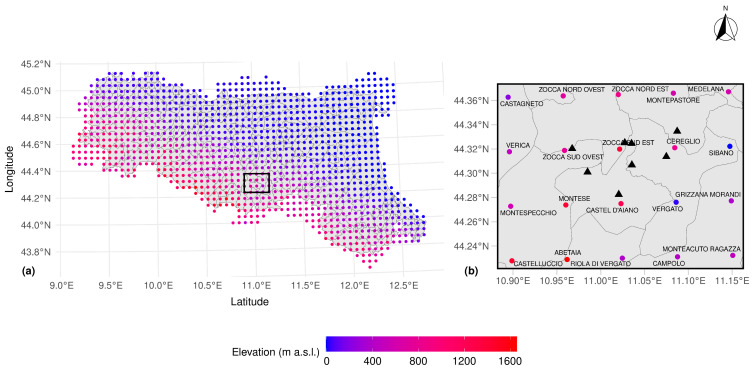
(**a**) Grid points of ERG5 “*Eraclito61*” Reanalysis Dataset throughout the Emilia-Romagna region, along with the distribution of absolute elevation of the nodes; (**b**) detail of the study area (black box) with the location and name of the 20 nodes involved in the analysis for SPEI assessment, the investigated springs (black triangles), and the boundaries of municipalities.

The assessment of aridity conditions across the study area was conducted through the calculation of the Standardized Precipitation–Evapotranspiration Index (SPEI) on a 3-monthly basis. The index was derived from precipitation and temperature data at 20 location nodes (Figure 2) within the ERG5 “*Eraclito61*” meteorological reanalysis dataset (https://dati.arpae.it/dataset/erg5-eraclito) (accessed on 19 April 2023). The ERG5 regular grid is built upon the data collected by the meteorological station network of the local Regional Authority of Emilia-Romagna (ARPAE). The dataset provides, for each node, daily records of air temperature (minimum and maximum) and precipitation, covering the period from 1961 to 2023 along a 5 km gridded square mesh (Figure 2), obtained by an adaptive interpolation technique that dissects temperature spatial variability through predictors like elevation, temperature lapse rate, and urbanization; precipitation was reconstructed taking into account topographic barriers and elevation differences through an enhanced Shepard interpolation algorithm [51].

**Figure 3:**
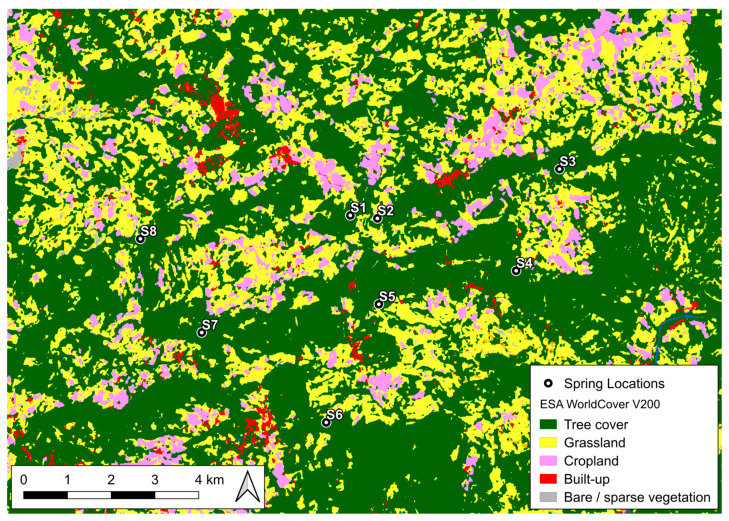
Land cover map of the investigated area (ESA WorldCover project based upon modified Copernicus 2021 Sentinel data processed by ESA WorldCover consortium; edited in QGIS3).

In order to delineate vegetation zones inside the investigated area, the ESA WorldCover 2021 v200 product was employed [52], providing a high-resolution (10 m) global land cover map derived from Sentinel-1 and Sentinel-2 data. It is presented in a latitude/longitude format according to the WGS 1984 ellipsoid, provided in 3 × 3-degree tiles in Cloud Optimized GeoTIFF format, classifying the global land surface into 11 land-cover categories. The following 5 occur in the investigated area: tree cover, grassland, cropland, built-up areas, and bare/sparse vegetation (Figure 3). Only continuous tree-covered areas (forest) were subjected to analysis.

The primary remote-sensing data for NDVI analysis were obtained from the Sentinel-2 satellite program (https://www.copernicus.eu/en/about-copernicus, accessed on 19 July 2023), a component of the European Space Agency’s Copernicus initiative. We used Level-2A surface-reflectance products (https://www.copernicus.eu/en/about-copernicus, accessed on 19 July 2023), which provide atmospherically corrected Bottom-of-Atmosphere (BOA) reflectance for 13 spectral bands at 10, 20, and 60 m spatial resolution. The NDVI was computed from the 10 m red (Band 4) and near-infrared (Band 8) bands. To remove cloud-contaminated observations, we employed the Sentinel-2 cloud-probability product available in the Google Earth Engine (COPERNICUS/S2_CLOUD_PROBABILITY). This layer is generated with the Sentinel-2 cloud-detector library based on a LightGBM model. For each Sentinel-2 Level-2A image from COPERNICUS/S2_SR_HARMONIZED, the corresponding cloud-probability image was joined, and a binary mask was created by excluding pixels with cloudProbability ≥ 60%. At the scene level, we also required CLOUDY_PIXEL_PERCENTAGE ≤ 10% to avoid images heavily affected by clouds. NDVI calculations were performed only on the remaining cloud-free pixels. The Scene Classification Layer (SCL) was not used for masking, and no additional cloud-shadow detection or dilation was applied. Although single-scene cloud shadows may appear as dark patches, their influence is strongly reduced by the long compositing period and the use of median aggregation because shadowed pixels do not recur at the same location across multiple acquisitions. Topographic effects were handled by relying on the harmonized Sentinel-2 Level-2A surface-reflectance product (S2_SR_HARMONIZED), which incorporates atmospheric correction and accounts for terrain elevation. No further BRDF or topographic normalization (e.g., C-correction, Minnaert’s correction) was applied. Remaining illumination differences in mountainous terrain are partially mitigated by: (i) using the NDVI, a ratio-based vegetation index less sensitive to multiplicative illumination effects, and (ii) the multi-temporal median composite, which reduces the influence of atypically dark or bright observations. Image selection covered five consecutive warm-season vegetation periods (May–September) for the years 2017–2021, thus encompassing both the major droughts of 2017 and 2021 and the period of monitored spring discharge (2020–2021). The number of available Level-2A images per year ranged between 24 and 35.

**Figure 4:**
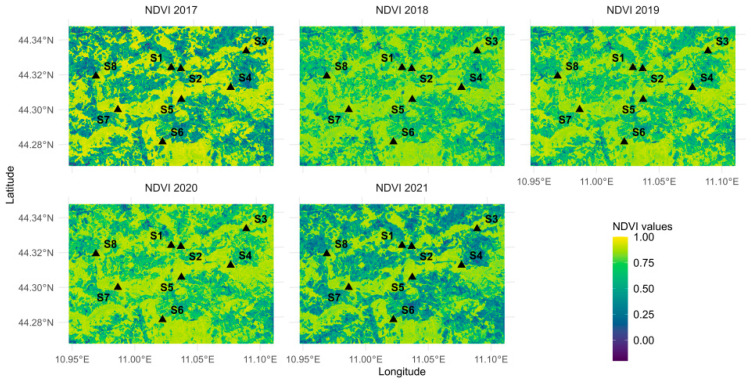
Annual median NDVI composite for years 2017–2021 relative to the period May–September (from Sentinel-2 Surface Reflectance data, 10 × 10 m spatial resolution, edited in R environment). Colorblind-friendly version prepared using the *viridis* palette.

NDVI values, from images available for each year, were processed within the Google Earth Engine [53] in order to generate a median annual NDVI composite. The composites, representative of each year, were extracted as a raster using a customized script developed in the R environment alongside a CSV file cataloging the image IDs, reprojected to the EPSG:32632 CRS (WGS 84/UTM zone 32N), and exported at a 10 × 10 m spatial resolution in GeoTIFF format (Figure 4).

**Figure 5:**
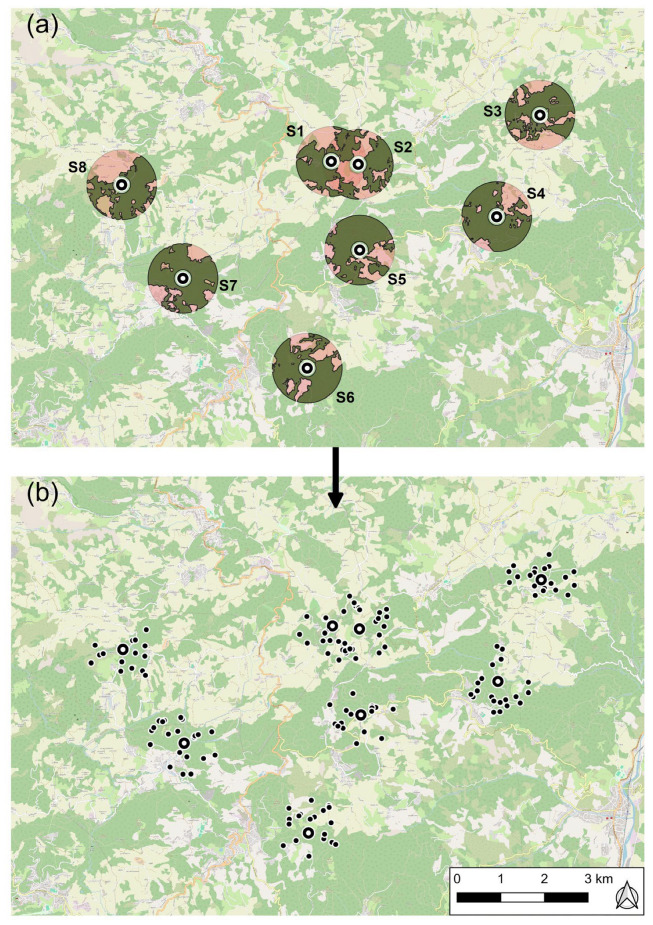
GIS-based process for counterpoint creation: (**a**) buffer zones centered on each spring with 200 m radius (inner circle) and 800 m radius (outer circle) located on land cover map in pale color, dark green zones represent the forest inside 800 m radius buffer zone; (**b**) random generation of counterpoints within the forested buffer zone.

An 800 m radius circular area (buffer zone) was created around each known spring location under study (Figure 5a). The inner 100 m radius of each buffer zone was considered as directly affected by spring discharge and low depth-to-water-table conditions, i.e., the NDVI for each spring was extracted in a 100 m radius around the spring point. Around each spring, we then defined a 200 m exclusion buffer. Using a point creation tool in QGIS (version 3.34.3; QGIS Development Team), a total of 15–21 random points were generated as counterpoints of the springs, inside each circular sector, at distances in the range of 200–800 m from each of the eight springs (Figure 5b). With this geometry, the 100 m NDVI disks around the counterpoints do not overlap with the 100 m NDVI disk (core discharge area) of the spring. The 200–800 m circular sector was clipped with the ESA WorldCover 2021 v200 “tree cover” class to keep only forest areas, and the QGIS random-point-generation tool placed control points randomly only inside the clipped forested areas (Figure 3). Regarding the apparent mismatch with Figure 5, where some random points appear to be located outside forested areas, this is due to employing, for a better graphical view, a more general OpenStreetMap basemap for land use.

These random points were chosen exclusively inside forested zones, as were the respective springs. Spatial points representing springs and control points were georeferenced in order to be aligned with the raster coordinate reference system of the NDVI. The number of counterpoints differs for each spring for the constraints derived from the extent of suitable forested area inside each circular sector.

**Figure 6:**
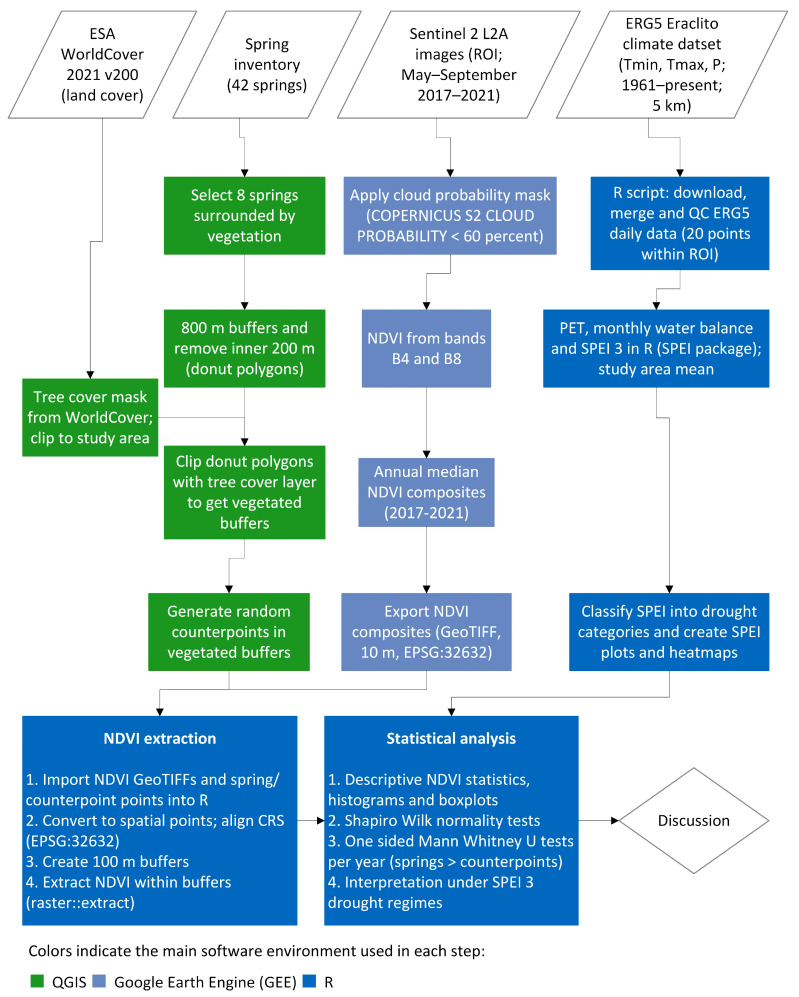
Overall workflow chart of the multi-stage procedure followed.

The complex multi-stage procedure followed is summarized in the overall workflow chart in Figure 6.

Monthly climate time series of minimum, maximum, mean air temperature (Tmin, Tmax, Tavg), and precipitation (P) were compiled for 20 sites within the study area covering the period 1961–2023. Data were quality-checked and aggregated to annual values, with mean annual temperature calculated as the average of monthly observations and annual precipitation computed as the sum of monthly totals. Long-term trends were estimated separately for each site using ordinary least squares (OLS) linear regression, with calendar year included as a predictor and centered on the first available year to facilitate interpretation of the intercept as a baseline value. Trend magnitudes were expressed as slopes per decade for clarity, and model performance was evaluated using the coefficient of determination (*R*^2^) and the statistical significance of the slope term. To assess whether rates of change differed among sites, models assuming a common temporal slope were compared with models including a year × site interaction using analysis of variance (ANOVA). All analyses were conducted in the R statistical environment, ensuring full reproducibility of the workflow.

Mann–Whitney comparisons were implemented using the R *wilcox.test* function. The function reports *W* (the rank-sum statistic) for the spring group; the corresponding *U* statistic is calculated as:

*U* = *W* − [n1 (n1 + 1)/2]

where n1 is the sample size of the spring group (n1 = 8). All *p*-values are one-sided (springs > controls) and were computed using the asymptotic approximation with continuity correction. Multiple comparison adjustments were performed using Holm’s method.

Finally, an explicit spring-level paired analysis was provided to avoid any risk of pseudoreplication due to spatial clustering of control points. Using annual median NDVI composites (2017–2021), NDVI was extracted within a 100 m buffer around each spring location. For each spring–year combination, the associated control counterpoints were summarized to a single background value (median NDVI; IQR also reported), yielding one paired comparison per spring and year. We then computed for ΔNDVI = NDVI spring − median (NDVI controls) for each spring (*n* = 8 per year), and tested whether ΔNDVI > 0 using a one-sided Wilcoxon signed-rank test.

## 3. Results

**Figure 7:**
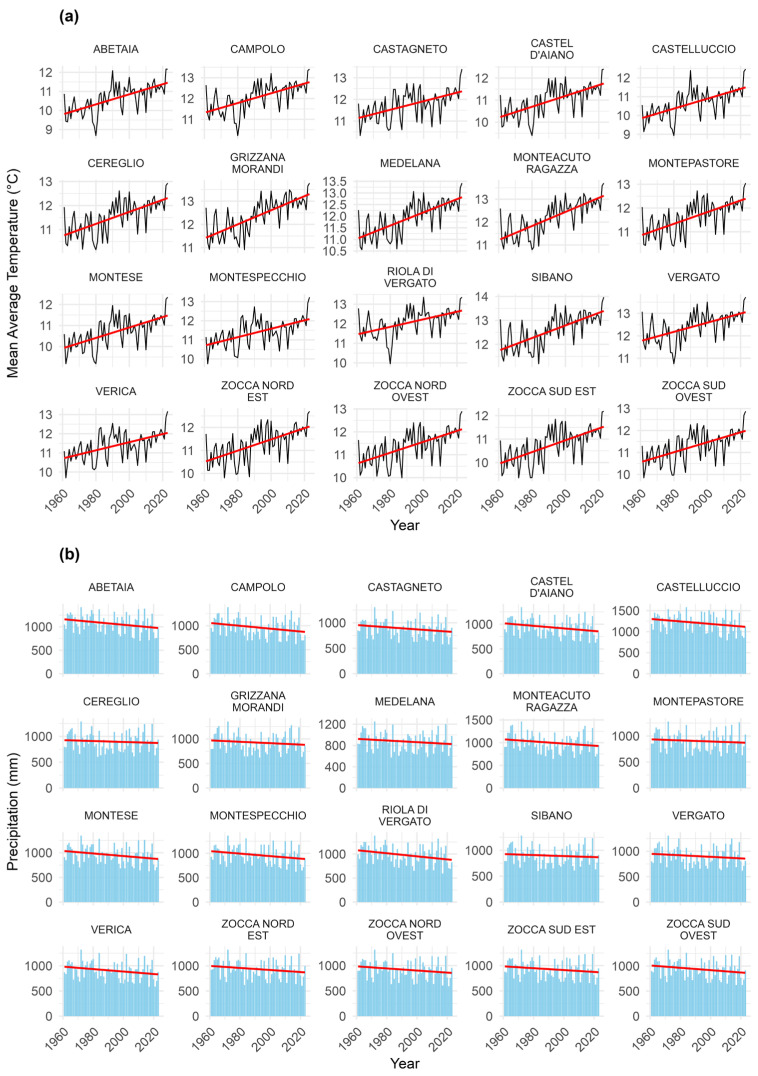
(**a**) Mean annual atmospheric temperature (°C) for the period 1961–2023; (**b**) total annual precipitation (mm) for the period 1961–2023. All graphs refer to the nodes of the ERG5 “*Eraclito61*” dataset shown in Figure 2. The red lines represent linear regression fits; corresponding regression equations, coefficients of determination (*R*^2^), and *p*-values are reported in Table 1 and Table 2.

The atmospheric temperature time series spanning from 1961 to 2023 shows a clear and consistent warming trend across all monitored locations (Figure 7a). Regression-based estimates (Table 1) indicate temperature trends ranging from 0.194 to 0.307 °C per decade, with the slope term displaying statistical significance (*p* < 0.05) at all 20 sites (Table 1). When extrapolated over the full study period (6.2 decades), these slopes correspond to a fitted increase in mean annual temperature (T_avg_) of approximately 1.2–1.9 °C, thereby quantitatively supporting the warming pattern observed considering the average yearly temperatures (T_avg_).

Precipitation time series exhibit an overall tendency toward a slight decline over the same period (Figure 7b). Regression slopes range from −32.04 to −8.85 mm per decade, with statistically significant trends detected at eight out of 20 sites (Table 2), providing a quantitative assessment of the precipitation decrease that was previously discussed in qualitative terms.

To assess whether the rate of change differs among sites, we compared a model assuming a common temporal trend across locations with a model allowing for site-specific slopes through a year × site interaction. For temperature, the interaction term is not significant (*F* = 0.63, *p* = 0.885), indicating that the warming rate is statistically homogeneous across the study area. Similarly, for precipitation, the interaction test is not significant (*F* = 0.31, *p* = 0.998), suggesting that precipitation trends are also statistically comparable among sites. Differences observed in the fitted intercepts reflect site-specific climatic baselines at the beginning of the record, and are attributable to geographic and topographic variability within the domain rather than to differences in temporal trends. Full regression outputs are provided to ensure transparency and reproducibility of both baseline conditions (intercepts) and temporal changes (slopes).

Given the consistent spatial behavior observed across monitoring locations, the SPEI was aggregated into a zonal average using all nodes of the “*Eraclito61*” grid, computed through the “SPEI” package [54] in R version 4.3.2 (R Core Team, 2023). The analysis was subsequently focused on the period from 2000 onwards to emphasize the more recent intensification and recurrence of drought events.

**Figure 8:**
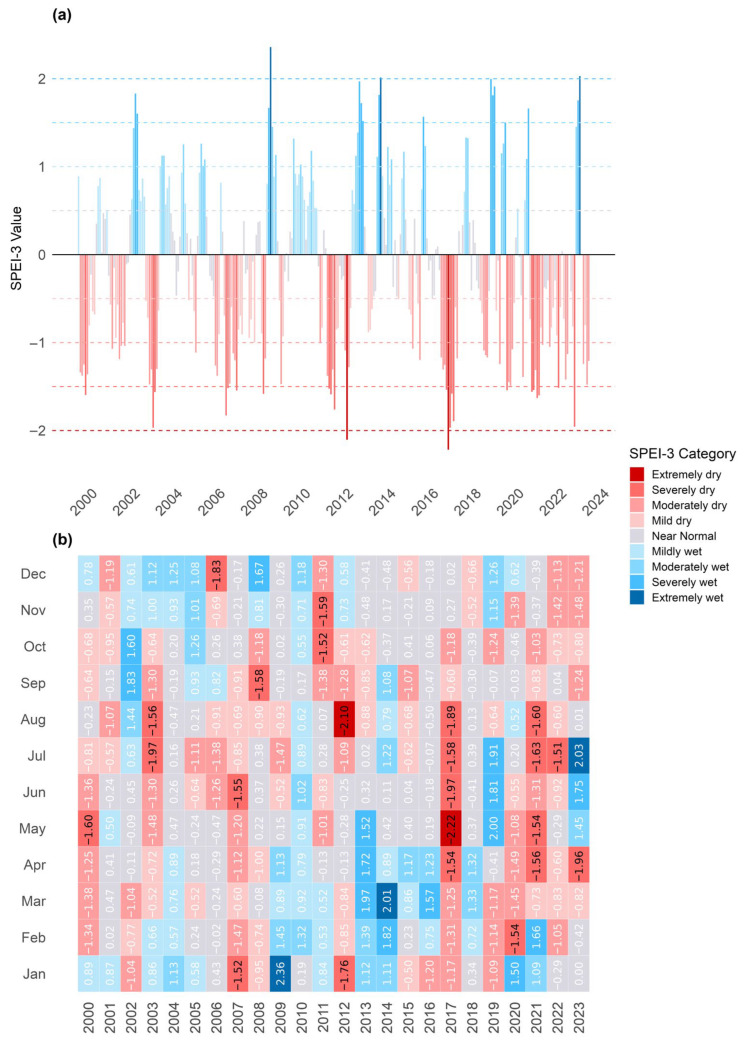
(**a**) Diagrammatic representation of SPEI-3 monthly median values; (**b**) SPEI-3 monthly median values. All data are derived from 20 nodes of “*Eraclito61*” grid dataset relative to the period 2000–2023 (located in Figure 2). The color scale is the same for both graphs.

The 3-month SPEI was categorized into levels ranging from “extremely dry” to “extremely wet” (Figure 8a,b). SPEI-3 was classified using the following cut-off points: extremely dry: SPEI-3 ≤ −2.0; severely dry: −2.0 < SPEI-3 ≤ −1.5; moderately dry: −1.5 < SPEI-3 ≤ −1.0; mildly dry: −1.0 < SPEI-3 ≤ −0.5; near normal: −0.5 < SPEI-3 ≤ 0.5; mildly wet: 0.5 < SPEI-3 ≤ 1.0; moderately wet: 1.0 < SPEI-3 ≤ 1.5; severely wet: 1.5 < SPEI-3 ≤ 2.0; extremely wet: SPEI-3 > 2.0. For the sake of simplification, in order to discriminate between “dry” and “wet” periods, the following grouped thresholds, consistent with the above ones, could be defined: severe drought months: SPEI-3 ≤ −1.5 (severely dry or worse); dry months: SPEI-3 ≤ −1.0 (moderately dry or worse); wet months: SPEI-3 ≥ +1.0 (moderately wet or better).

The data highlight several significant drought periods, particularly in the years 2003, 2007, 2012, 2017, and 2021–2022 (Figure 8a). For instance, May 2017 and August 2012 experienced “severely dry” conditions, with SPEI values of −2.22 and −2.10, respectively, marking some of the most acute droughts within the dataset. As observable in Figure 8b, the years 2017 and 2021 experienced the most consecutive months of dryness within the investigated timeframe; for both years, five severe dry months were recorded, according to the simplified ranking, from April to August (with the exception of June in 2021) during the vegetative period.

Using the R environment’s “*raster::extract*” function, NDVI values were extracted for a defined 100 m buffer zone surrounding each spatial point and each counterpoint, enabling a relative comparison of the vegetation between the discharge area (springs) and more distant portions (counterpoints) with the same land use (continuous forest). A major role was assigned to the median value to reduce outlier effects. Having chosen the counterpoints at a distance higher than 200 m from each spring, there is no overlap between the NDVI 100 m buffer zone relative to the spring and to the respective counterpoints, so the NDVI relative to the counterpoints is not affected by the 100 m radius discharge zone.

**Figure 9:**
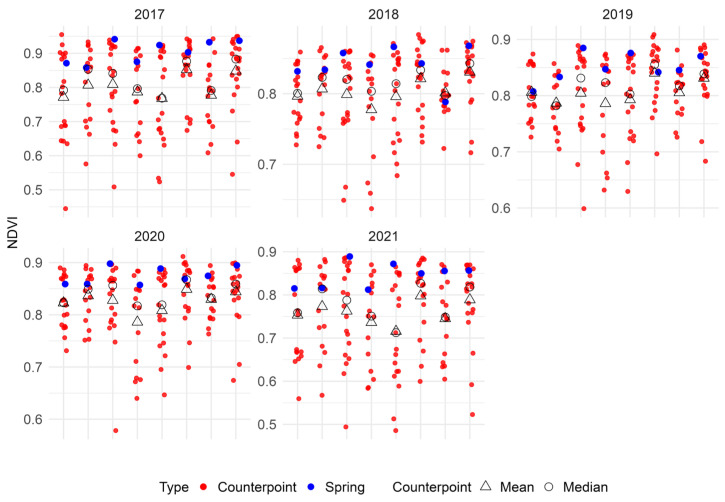
Distribution of median NDVI values, relatively to a 100 m radius circular zone centered on the springs investigated (blue dots) and on control points (red dots) for each of the five years of investigation. The mean and median values of NDVI for all counterpoints associated to a certain spring are indicated.

During the 2017–2021 time span, the median NDVI in spring points consistently exhibited a higher value compared to counterpoints, with a median range between 0.91 (2017) and 0.84 (2018) (Figure 9; Table 3). Control points displayed considerable variability over the study period, in contrast to spring points. Notably, 2021 marked the lowest median NDVI value (0.79).

The Shapiro–Wilk test [55] was utilized to assess the normality of NDVI distribution for spring points and counterpoints (Table 4). The results indicate that for the spring areas, NDVI values generally do not significantly deviate from a normal distribution. Conversely, for the counterpoints, there is a consistent pattern of significant departure from normality across all years, with extremely low *p*-values, indicating non-normal distributions.

Given the frequent non-normal distribution of NDVI values, particularly for the counterpoints, and the limited sample size, the Mann–Whitney *U* test (nonparametric) was chosen in order to compare median values between the two groups, verifying their independence [56]. Individual *U* tests were performed for each year, between 2017 and 2021, comparing the NDVI value of eight spring points with those of the respective 145 counterpoints. The null hypothesis, which states that the median NDVI values of the spring areas are less than or equal to those of the counterpoints, was tested in a one-sided way. The results, reported in Table 5, strongly support the hypothesis that spring areas exhibit statistically significant, higher median NDVI levels than counterpoints, as expressed by the associated *p*-value that is always lower than 5 × 10^−2^.

The contrast in the NDVI was most pronounced during SPEI-defined dry years, with the highest effect size observed in 2017 (rrb = 0.497, Holm’s *p* = 0.020), supporting the hypothesis that springs act as critical green refugia during water-stressed periods (Table 6).

**Figure 10:**
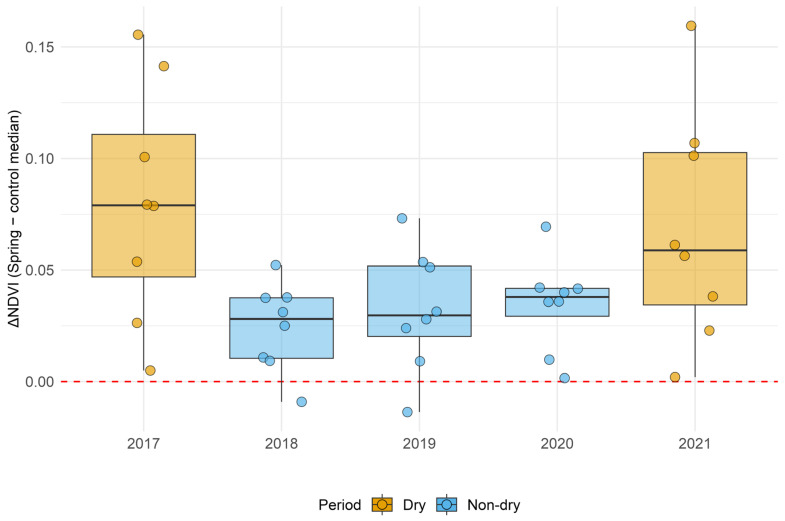
ΔNDVI for dry and non-dry years defined by means of the SPEI-based drought classification.

The paired differences are visualized in Figure 10 (ΔNDVI by year), and the full Wilcoxon outputs (*n*, *V*, one-sided *p*, median ΔNDVI, and *Holm-adjusted p*-values across years) are reported in Table 7. Across all years, ΔNDVI is predominantly positive and statistically greater than zero, supporting the statement that vegetation in spring discharge areas is consistently greener than its local background.

## 4. Discussion

The analysis of time series of precipitation and atmospheric temperature, along with the associated SPEI value pattern, provides evidence that the investigated area—located at the northern outer rim of the Mediterranean zone—has experienced an uptick in frequency and severity of summer droughts since the beginning of the century, generally anticipated by reduced precipitation and higher average temperatures during the recharge season (November–April). The drought pattern seems to follow a five-year cycle, with notable dry spells in 2012, 2017, and 2021–2022, confirming what has been evidenced in the whole of southern Europe [5,57,58,59,60].

Over the five growing seasons from 2017 to 2021, NDVI analysis demonstrated a general stability, especially during relatively wet periods, but a marked decrease in the median value occurred in 2021 (an NDVI value equal to 0.8527), reflecting the associated verified impact of drought conditions, evidenced by the very low SPEI values.

Comparing NDVI values between spring areas and their corresponding control points during drought periods highlights a clear contrast in vegetation dynamics. Spring areas consistently maintained higher NDVI values throughout the study period, reflecting healthier and more resilient vegetation cover. Although both spring areas and control points exhibited temporal fluctuations, the decline observed at the control points was noticeably more pronounced, suggesting greater exposure to environmental stressors.

Several factors could theoretically influence NDVI behavior at spring discharge outlets, including the depth to the water table, vegetation type, and geological structure. However, the variability of these factors among the selected springs is negligible relative to the differences between spring points and their associated control areas. All eight investigated springs are located at the contact between the arenitic aquifer unit of the *Pantano* Formation and adjacent low-permeability aquitards, as clearly shown in Figure 1 (bottom). This contact represents not only a geological contrast but also a marked morphological transition. The arenitic outcrops (brown areas in Figure 1) are characterized by steep slopes, typically ranging from 30% to 45%, whereas the more clay-rich aquitards, which are more susceptible to erosion, occur in gentler terrain with slopes of approximately 10–20%.

Groundwater flow is restricted to the aquifer unit, as the aquitards do not sustain active groundwater circulation. At the spring outlets, the depth to the water table is consistently shallow (approximately 1–2 m below ground surface), as directly observed within the spring-boxes where groundwater discharge occurs. Moving away from the spring outlets within the aquifer, a substantial increase in depth to the water table is expected. Direct measurements from boreholes in the *Pantano* Formation indicate hydraulic gradients of 5–10% [7,61], which are significantly lower than surface slopes. Consequently, even in the absence of boreholes near the control points, a pronounced deepening of the water table is anticipated beyond the 200 m buffer zone around each spring outlet. Within the aquifer outcrop, only the immediate discharge area is characterized by a very shallow water table. Therefore, variations in water-table depth do not meaningfully contribute to NDVI differences among the spring points, which all exhibit similar groundwater conditions, whereas the greater distance of the water table from the root zone in the surrounding forested areas is the primary control on reduced NDVI values.

Vegetation composition in the forested areas surrounding the springs is also homogeneous. Springs influenced by agricultural land or urban features were deliberately excluded. The selected buffer zones are consistently dominated by oak and hornbeam forests, with scattered chestnut groves. As a result, vegetation type does not contribute significantly to NDVI variability among the eight spring sites.

The geological setting is equally uniform. All springs discharge from the same lithostratigraphic unit—the *Pantano* Formation—composed of relatively homogeneous fractured arenite. Discharge occurs where this unit contacts lower-permeability aquitards, creating a consistent hydraulic threshold. Given the shared lithology and structural conditions, geological heterogeneity cannot explain the observed NDVI differences. Accordingly, the eight spring points can be considered a homogeneous population when compared with the control points, where depth to the water table increases sharply and exerts the dominant control on NDVI decline, as supported by the collected data.

For the randomly selected control points, vegetation stress during dry periods is the dominant factor influencing NDVI, outweighing potential topographic effects such as elevation, slope, or aspect. Elevation differences between spring points (543–714 m a.s.l.) and control points are modest; on average, control points are approximately 200 m higher than the corresponding spring outlets, a difference insufficient to produce NDVI variations comparable to those induced by summer water stress. Slope values are relatively uniform across the *Pantano* Formation outcrops due to the lithological homogeneity of the unit, unlike layered turbiditic formations that exhibit greater morphological variability. The aspect varies among control points; however, because control points were selected randomly in all directions around each spring (Figure 5), the full range of aspect variability is adequately represented within the population of control points.

The resilience assessment through the comparison of NDVI values near spring areas and the 145 control points provided critical insights into the ecosystem’s response to drought conditions. Mann–Whitney U tests supported the significance of disparities in NDVI values between spring areas and counterpoints. Higher NDVI values near springs compared to points far away from springs are statistically consistent and relevant, suggesting that vegetation in these areas maintained better health and greenness during drought periods, likely due to groundwater support as a consequence of the proximity of the water table to the ground surface. The greater range in NDVI values observed at counterpoints, especially during dry periods, can be attributed to the blunt difference in species composition and vegetation dynamics when compared to spring areas. On the other hand, during wet periods, the NDVI values for counterpoints and springs were similar, indicating the mitigating effects of increased moisture availability on vegetation health. Springs are relevant spots sustaining ecosystem resilience, with a substantial “activation” of point-type terrestrial groundwater-dependent ecosystems during drought events. The relevance of spring locations in affecting tree growth, acting as a valuable ecological buffer, has been demonstrated on ponderosa pines in the southwestern United States [62].

The findings indicate the feasibility of leveraging the NDVI to detect vegetation anomalies associated with groundwater discharge within naturally vegetated landscapes. This is particularly valuable given that the NDVI can be derived from widely available remote-sensing platforms, providing high-resolution and repeatable observations. Accordingly, the proposed approach supports the use of the NDVI as an indicator of groundwater discharge likelihood, based on spatially explicit vegetation-condition data obtained from satellite imagery. While not a deterministic tool for locating springs, this method offers a practical means of highlighting areas where groundwater–ecosystem interactions are most probable.

## 5. Conclusions

This study investigated the resilience of natural vegetation surrounding groundwater discharge outlets (springs) in a Mediterranean mountainous region (Northern Apennines, Italy) during drought periods, contextualized through the Standardized Precipitation–Evapotranspiration Index (SPEI), using the Normalized Difference Vegetation Index (NDVI) derived from Sentinel-2 imagery. The accurate geolocation of spring outlets within fully forested environments ensured the reliability of the spatial comparison framework and strengthened the robustness of the adopted methodological approach.

Statistically significant differences in NDVI values between spring-centered areas and control points were demonstrated during SPEI-identified drought periods, with land cover held constant. These results highlight the role of proximity to groundwater discharge in sustaining vegetation vigor under climatic stress. The observed resilience of vegetation near springs, compared to surrounding areas, emphasizes the buffering function of groundwater during dry conditions.

The findings confirm the applicability of the NDVI for assessing vegetation responses to groundwater discharge in tapped springs and within a mesic, middle-latitude climatic context. While the approach does not deterministically identify unknown springs, it highlights localized vegetation anomalies that may indicate groundwater discharge zones under well-constrained geological and environmental conditions. This capability has practical implications for supporting spring reconnaissance, groundwater monitoring, and drought-related vegetation assessment in remote mountainous regions.

Future developments could include the integration of advanced data-driven approaches, such as artificial intelligence techniques, to systematically analyze gridded SPEI and NDVI datasets and enhance the scalability and operational implementation of the method.

## Figures and Tables

**Table 1:** Linear regression statistics for mean annual temperature trends (1961–2023).

Site	Regression Equation	Slope (°C Dec^−1^)	*R* ^2^	*p*-Value
ABETAIA	Tavg = 9.82 + 0.0264·(Year − 1961)	0.264	0.406	<1.0 × 10^−10^
CAMPOLO	Tavg = 11.34 + 0.0233·(Year − 1961)	0.233	0.376	1.0 × 10^−7^
CASTAGNETO	Tavg = 11.14 + 0.0198·(Year − 1961)	0.198	0.285	6.6 × 10^−6^
CASTEL D’AIANO	Tavg = 10.23 + 0.0245·(Year − 1961)	0.245	0.402	<1.0 × 10^−10^
CASTELLUCCIO	Tavg = 9.86 + 0.0263·(Year − 1961)	0.263	0.393	<1.0 × 10^−10^
CEREGLIO	Tavg = 10.76 + 0.0250·(Year − 1961)	0.250	0.418	<1.0 × 10^−10^
GRIZZANA MORANDI	Tavg = 11.42 + 0.0303·(Year − 1961)	0.303	0.531	<1.0 × 10^−10^
MEDELANA	Tavg = 11.05 + 0.0284·(Year − 1961)	0.284	0.491	<1.0 × 10^−10^
MONTEACUTO RAGAZZA	Tavg = 11.25 + 0.0307·(Year − 1961)	0.307	0.548	<1.0 × 10^−10^
MONTEPASTORE	Tavg = 10.87 + 0.0246·(Year − 1961)	0.246	0.402	<1.0 × 10^−10^
MONTESE	Tavg = 9.94 + 0.0248·(Year − 1961)	0.248	0.384	1.0 × 10^−7^
MONTESPECCHIO	Tavg = 10.72 + 0.0221·(Year − 1961)	0.221	0.313	1.9 × 10^−6^
RIOLA DI VERGATO	Tavg = 11.47 + 0.0194·(Year − 1961)	0.194	0.267	1.5 × 10^−5^
SIBANO	Tavg = 11.77 + 0.0263·(Year − 1961)	0.263	0.469	<1.0 × 10^−10^
VERGATO	Tavg = 11.79 + 0.0204·(Year − 1961)	0.204	0.325	1.1 × 10^−6^
VERICA	Tavg = 10.73 + 0.0213·(Year − 1961)	0.213	0.290	5.3 × 10^−6^
ZOCCA NORD EST	Tavg = 10.53 + 0.0244·(Year − 1961)	0.244	0.376	1.0 × 10^−7^
ZOCCA NORD OVEST	Tavg = 10.63 + 0.0239·(Year − 1961)	0.239	0.370	1.0 × 10^−7^
ZOCCA SUD EST	Tavg = 9.97 + 0.0251·(Year − 1961)	0.251	0.395	<1.0 × 10^−10^
ZOCCA SUD OVEST	Tavg = 10.58 + 0.0227·(Year − 1961)	0.227	0.349	3.0 × 10^−7^

**Table 2:** Linear regression statistics for annual precipitation trends (1961–2023).

Site	Regression Equation	Slope (mm Dec^−1^)	*R* ^2^	*p*-Value
ABETAIA	P = 1159.3 − 2.986·(Year − 1961)	−29.86	0.081	2.4 × 10^−2^
CAMPOLO	P = 1054.2 − 2.976·(Year − 1961)	−29.76	0.078	2.7 × 10^−2^
CASTAGNETO	P = 954.4 − 2.135·(Year − 1961)	−21.35	0.051	7.4 × 10^−2^
CASTEL D’AIANO	P = 1016.6 − 2.604·(Year − 1961)	−26.04	0.068	3.8 × 10^−2^
CASTELLUCCIO	P = 1300.1 − 2.977·(Year − 1961)	−29.77	0.069	3.8 × 10^−2^
CEREGLIO	P = 926.4 − 0.885·(Year − 1961)	−8.85	0.008	4.8 × 10^−1^
GRIZZANA MORANDI	P = 968.6 − 1.424·(Year − 1961)	−14.24	0.019	2.8 × 10^−1^
MEDELANA	P = 922.9 − 1.567·(Year − 1961)	−15.67	0.026	2.1 × 10^−1^
MONTEACUTO RAGAZZA	P = 1071.0 − 2.338·(Year − 1961)	−23.38	0.044	9.8 × 10^−2^
MONTEPASTORE	P = 935.7 − 1.032·(Year − 1961)	−10.32	0.011	4.2 × 10^−1^
MONTESE	P = 1035.4 − 2.605·(Year − 1961)	−26.05	0.067	4.0 × 10^−2^
MONTESPECCHIO	P = 1044.0 − 2.578·(Year − 1961)	−25.78	0.066	4.2 × 10^−2^
RIOLA DI VERGATO	P = 1076.9 − 3.204·(Year − 1961)	−32.04	0.090	1.7 × 10^−2^
SIBANO	P = 926.2 − 0.978·(Year − 1961)	−9.78	0.010	4.3 × 10^−1^
VERGATO	P = 949.3 − 1.510·(Year − 1961)	−15.10	0.023	2.4 × 10^−1^
VERICA	P = 980.8 − 2.438·(Year − 1961)	−24.38	0.063	4.7 × 10^−2^
ZOCCA NORD EST	P = 996.8 − 2.035·(Year − 1961)	−20.35	0.042	1.1 × 10^−1^
ZOCCA NORD OVEST	P = 983.6 − 2.066·(Year − 1961)	−20.66	0.045	9.6 × 10^−2^
ZOCCA SUD EST	P = 984.5 − 1.849·(Year − 1961)	−18.49	0.036	1.4 × 10^−1^
ZOCCA SUD OVEST	P = 1008.4 − 2.332·(Year − 1961)	−23.32	0.056	6.2 × 10^−2^

**Table 3:** Summary of descriptive statistics for NDVI values in the springs and counterpoints’ 100 m buffer zones during the study period.

Year	Type	*Min*	*First Quartile*	*Median*	*Mean*	*Third Quartile*	*Max*
2017	Spring	0.86	0.87	0.91	0.91	0.93	0.94
2017	Counter	0.44	0.70	0.84	0.80	0.90	0.95
2018	Spring	0.79	0.83	0.84	0.84	0.86	0.87
2018	Counter	0.64	0.77	0.82	0.80	0.85	0.88
2019	Spring	0.81	0.84	0.85	0.85	0.87	0.88
2019	Counter	0.60	0.77	0.82	0.81	0.86	0.91
2020	Spring	0.86	0.86	0.87	0.87	0.89	0.90
2020	Counter	0.58	0.79	0.85	0.83	0.87	0.91
2021	Spring	0.81	0.82	0.85	0.85	0.86	0.89
2021	Counter	0.49	0.67	0.79	0.76	0.85	0.89

**Table 4:** Summary of Shapiro–Wilk test results of NDVI values in the springs and counterpoints’ 100 m buffer zones.

Springs			Counterpoints	
Year	*W*	*p-Value*	*W*	*p-Value*
2017	0.883	2.0 × 10^−1^	0.911	8.7 × 10^−8^
2018	0.881	1.9 × 10^−1^	0.927	9.1 × 10^−7^
2019	0.955	7.6 × 10^−1^	0.932	1.9 × 10^−6^
2020	0.874	1.6 × 10^−1^	0.890	6.0 × 10^−9^
2021	0.901	3.0 × 10^−1^	0.909	6.9 × 10^−8^

**Table 5:** Summary of Mann–Whitney *U* test results.

Year	*U Statistics*	*p Value*
2017	904	4.0 × 10^−3^
2018	828	2.1 × 10^−2^
2019	805	3.3 × 10^−2^
2020	863	1.0 × 10^−2^
2021	861	1.1 × 10^−2^

**Table 6:** Median NDVI and interquartile range (IQR) values for springs (*n* = 8) and controls (*n* = 145) for each year. The Mann–Whitney test statistics (*W*, and *U* derived from *W*), one-sided *p*-values, Holm-adjusted *p*-values across years, and an effect size (rank-biserial correlation). Years classified as “dry” follow the SPEI-based drought classification described in Figure 8; the remaining years are reported as non-dry.

*Year*	*Period*	*Spring Median [IQR]*	*Control Median [IQR]*	*W*	*U*	*p-Value*	*p (Holm)*	*rrb*
*2017*	Dry	0.914 [0.874, 0.934]	0.840 [0.701, 0.903]	904	868	0.004	0.020	0.497
*2018*	Non-dry	0.842 [0.833, 0.860]	0.821 [0.772, 0.846]	828	792	0.021	0.043	0.366
*2019*	Non-dry	0.846 [0.840, 0.872]	0.821 [0.767, 0.858]	805	769	0.033	0.043	0.326
*2020*	Non-dry	0.872 [0.859, 0.890]	0.847 [0.792, 0.875]	863	827	0.010	0.041	0.426
*2021*	Dry	0.853 [0.816, 0.860]	0.791 [0.668, 0.851]	861	825	0.011	0.041	0.422

**Table 7:** Full Wilcoxon outputs (*n*, *V*, one-sided *p*, median ΔNDVI, and *Holm-adjusted p*-values across years).

*Year*	*n*	*V*	*p_Value*	*Median ΔNDVI*	*p_Holm*
2017	8	36	3.9 × 10^−3^	0.079	2.0 × 10^−2^
2018	8	35	7.8 × 10^−3^		2.0 × 10^−2^
2019	8	34	1.2 × 10^−2^	0.028	2.0 × 10^−2^
2020	8	36	3.9 × 10^−3^		2.0 × 10^−2^
2021	8	36	3.9 × 10^−3^	0.030	2.0 × 10^−2^

## Data Availability

The original contributions presented in this study are included in the article. Further inquiries can be directed to the corresponding author.

## Abbreviations

The following abbreviations are used in this manuscript:

NDVI	Normalized Difference Vegetation Index
PAT	Pantano Formation
SPEI	Standardized Precipitation–Evapotranspiration Index

## References

[1] Glazier D.S., Likens G.E. (2005). Springs. Encyclopedia of Inland Waters.

[2] Amanambu A.C., Obarein O.A., Mossa J., Li L., Ayeni S.S., Balogun O., Oyebamiji A., Ochege F.U. (2020). Groundwater system and climate change: Present status and future considerations. J. Hydrol..

[3] Atawneh D.A., Cartwright N., Bertone E. (2021). Climate change and its impact on the projected values of groundwater recharge: A review. J. Hydrol..

[4] Filippini M., Amorosi A., Dinelli E., Segadelli S., Landi L., Casati T., Gargini A. (2025). Depositional environment of shallow-marine arenites in the Northern Apennines (Italy) affects aquifer performance: An interpretive key to groundwater management in a climate change scenario. J. Hydrol. Reg. Stud..

[5] Riedel T., Weber T.K.D. (2020). Review: The influence of global change on Europe’s water cycle and groundwater recharge. Hydrogeol. J..

[6] Taylor R.G., Scanlon B., Döll P., Rodell M., van Beek R., Wada Y., Longuevergne L., Leblanc M., Famiglietti J.S., Edmunds M. (2013). Ground water and climate change. Nat. Clim. Change.

[7] Filippini M., Segadelli S., Dinelli E., Failoni M., Stumpp C., Vignaroli G., Casati T., Tiboni B., Gargini A. (2024). Hydrogeological assessment of a major spring discharging from a calcarenitic aquifer with implications on resilience to climate change. Sci. Total Environ..

[8] IPCC (Intergovernmental Panel on Climate Change) (2023). Climate Change 2023: Synthesis Report.

[9] Li H., Li Z., Chen Y., Xiang Y., Liu Y., Kayumba P.M., Li X. (2021). Drylands face potential threat of robust drought in the CMIP6 SSPs scenarios. Environ. Res. Lett..

[10] Spinoni J., Vogt J.V., Naumann G., Barbosa P., Dosio A. (2018). Will drought events become more frequent and severe in Europe?. Int. J. Climatol..

[11] Vicente-Serrano S.M., Lopez-Moreno J.I., Beguería S., Lorenzo-Lacruz J., Sanchez-Lorenzo A., García-Ruiz J.M., Azorin-Molina C., Morán-Tejeda E., Revuelto J., Trigo R. (2014). Evidence of increasing drought severity caused by temperature rise in southern Europe. Environ. Res. Lett..

[12] European Drought Observatory What is Drought. 2023 JRC European Commission. https://edo.jrc.ec.europa.eu/edov2/php/index.php?id=1001.

[13] Cook B.I., Mankin J.S., Anchukaitis K.J. (2018). Climate change and drought: From past to future. Curr. Clim. Change Rep..

[14] Naumann G., Alfieri L., Wyser K., Mentaschi L., Betts R.A., Carrao H., Spinoni J., Vogt J., Feyen L. (2018). Global changes in drought conditions under different levels of warming. Geophys. Res. Lett..

[15] Vicente-Serrano S.M., Beguería S., López-Moreno J.I. (2010). A Multiscalar Drought Index sensitive to Global Warming: The Standardized Precipitation Evapotranspiration Index. J. Clim..

[16] Hargreaves G.H., Samani Z.A. (1985). Reference crop evapotranspiration from temperature. Appl. Eng. Agric..

[17] Li B., Zhou W., Zhao Y., Ju Q., Yu Z., Liang Z., Acharya K. (2015). Using the SPEI to assess recent climate change in the Yarlung Zangbo River Basin, South Tibet. Water.

[18] EL-Vilaly M.A.S., Didan K., Marsh S.E., Crimmins M.A., Munoz A.B. (2018). Characterizing drought effects on vegetation productivity in the Four Corners region of the US Southwest. Sustainability.

[19] McDowell N., Pockman W.T., Allen C.D., Breshears D.D., Cobb N., Kolb T., Plaut J., Sperry J., West A., Williams D.G. (2008). Mechanisms of plant survival and mortality during drought: Why do some plants survive while others succumb to drought?. New Phytol..

[20] Dahm C.N., Baker M.A., Moore D.I., Thibault J.R. (2003). Coupled biogeochemical and hydrological responses of streams and rivers to drought. Freshw. Biol..

[21] Wang S., Li R., Wu Y., Zhao S. (2022). Effects of multi-temporal scale drought on vegetation dynamics in Inner Mongolia from 1982 to 2015, China. Ecol. Indic..

[22] Brauman K.A. (2015). Hydrologic ecosystem services: Linking ecohydrologic processes to human well-being in water research and watershed management. Wiley Interdiscip. Rev. Water.

[23] Chang H., Bonnette M.R. (2016). Climate change and water related ecosystem services: Impacts of drought in California, USA. Ecosyst. Health Sustain..

[24] Jaeger W.K., Plantinga A.J., Chang H., Dello K., Grant G., Hulse D., McDonnell J.J., Lancaster S., Moradkhani H., Morzillo A.T. (2013). Toward a formal definition of water scarcity in natural-human systems. Water Resour. Res..

[25] Sun G., Wei X., Hao L., Sanchis M.G., Hou Y., Yousefpour R., Tang R., Zhang Z. (2023). Forest hydrology modeling tools for watershed management: A review. For. Ecol. Manag..

[26] Peven G., Eitel J.U.H., Link T.E., Estey E.W., Engels M. (2025). The role of spring ecosystems as climate refugia in a semi-arid environment. Ecohydrology.

[27] Bertrand G., Masini J., Goldscheider N., Meeks J., Lavastre V., Celle-Jeanton H., Gobat J.-M., Hunkeler D. (2014). Determination of spatiotemporal variability of tree water uptake using stable isotopes (δ^18^O, δ^2^H) in an alluvial system supplied by a high-altitude watershed, Pfyn forest, Switzerland. Ecohydrology.

[28] Brolsma R.J., van Vliet M.T.H., Bierkens M.F.P. (2010). Climate change impact on a groundwater-influenced hillslope ecosystem. Water Resour. Res..

[29] Ehleringer J.R., Dawson T.E. (1992). Water uptake by plants: Perspectives from stable isotope composition. Plant Cell Environ..

[30] Fan Y., Li H., Miguez-Macho G. (2013). Global patterns of groundwater table depth. Science.

[31] Lowry C.S., Loheide S.P. (2010). Groundwater-dependent vegetation: Quantifying the groundwater subsidy. Water Resour. Res..

[32] Kløve B., Ala-Aho P., Bertrand G., Gurdak J.J., Kupfersberger H., Kværner J., Muotka T., Mykrä H., Preda E., Rossi P. (2014). Climate change impacts on groundwater and dependent ecosystems. J. Hydrol..

[33] Maxwell R.M., Kollet S.J. (2008). Interdependence of groundwater dynamics and land-energy feedbacks under climate change. Nat. Geosci..

[34] Soylu M.E., Kucharik C.J., Loheide S.P. (2014). Influence of groundwater on plant water use and productivity: Development of an integrated ecosystem–variably saturated soil water flow model. Agric. For. Meteorol..

[35] Rimkus E., Stonevicius E., Kilpys J., Maciulyte V., Valiukas D. (2017). Drought identification in the eastern Baltic region using NDVI. Earth Syst. Dyn..

[36] NASA Earth Observatory (2000). Measuring Vegetation (NDVI & EVI). https://earthobservatory.nasa.gov/features/MeasuringVegetation/measuring_vegetation_2.php.

[37] Pettorelli N., Vik J.O., Mysterud A., Gaillard J.M., Tucker C.J., Stenseth N.C. (2005). Using the satellite-derived NDVI to assess ecological responses to environmental change. Trends Ecol. Evol..

[38] Aguilar C., Zinnert J.C., Polo M.J., Young D.R. (2012). NDVI as an indicator for changes in water availability to woody vegetation. Ecol. Indic..

[39] Wang J., Rich P.M., Price K.P. (2003). Temporal responses of NDVI to precipitation and temperature in the central Great Plains, USA. Int. J. Remote Sens..

[40] Cartwright J., Johnson H.M. (2018). Springs as Hydrologic Refugia in a Changing Climate? A Remote-Sensing Approach. Ecosphere.

[41] Ashraf A., Ali M. (2025). Appraisal of spring distribution across rain-fed plateau of Pakistan for sustainable development. Discov. Water.

[42] Baronetti A., González Hidalgo J.C., Vicente Serrano S.M., Acquaotta F., Fratianni S. (2020). A weekly spatio temporal distribution of drought events over the Po Plain (North Italy) in the last five decades. Int. J. Climatol..

[43] Cervi F., Petronici F., Castellarin A., Marcaccio M., Bertolini A., Borgatti L. (2018). Climate-change potential effects on the hydrological regime of freshwater springs in the Italian Northern Apennines. Sci. Total Environ..

[44] Baronetti A., Menichini M., Provenzale A. (2024). Vegetation response to droughts: The case of northern Italy. Int. J. Climatol..

[45] Boccaletti M., Corti G., Martelli L. (2011). Recent and active tectonics of the external zone of the Northern Apennines (Italy). Int. J. Earth Sci..

[46] Carminati E., Doglioni C. (2012). Alps vs. Apennines: The paradigm of a tectonically asymmetric Earth. Earth-Sci. Rev..

[47] Amorosi A. (1996). Miocene shallow-water deposits of the northern Apennines: A stratigraphic marker across a dominantly turbidite foreland-basin succession. Neth. J. Geosci..

[48] Kottek M., Grieser J., Beck C., Rudolf B., Rubel F. (2006). World Map of the Köppen-Geiger climate classification updated. Meteorol. Z..

[49] Gargini A., Vincenzi V., Piccinini L., Zuppi G.M., Canuti P. (2008). Groundwater flow systems in turbidites of Northern Apennines (Italy): Natural discharge and high speed railway tunnels drainage. Hydrogeol. J..

[50] Segadelli S., Filippini M., Monti A., Celico F., Gargini A. (2021). Estimation of recharge in mountain hard-rock aquifers based on discrete spring discharge monitoring during base-flow recession. Hydrogeol. J..

[51] Antolini G., Auteri L., Pavan V., Tomei F., Tomozeiu R., Marletto V. (2016). A daily high-resolution gridded climatic data set for Emilia-Romagna, Italy, during 1961–2010. Int. J. Climatol..

[52] Zanaga D., Van De Kerchove R., Daems D., De Keersmaecker W., Brockmann C., Kirches G., Wevers J., Cartus O., Santoro M., Fritz S. ESA WorldCover 10 m 2021 v200, Version v200. 2022. Zenodo. https://zenodo.org/records/7254221.

[53] Gorelick N., Hancher M., Dixon M., Ilyushchenko S., Thau D., Moore R. (2017). Google Earth Engine: Planetary-scale geospatial analysis for everyone. Remote Sens. Environ..

[54] Beguería S., Vicente-Serrano S.M. (2023). SPEI: Calculation of the Standardized Precipitation-Evapotranspiration Index. Technical Report. https://cran.r-project.org/web/packages/SPEI/SPEI.pdf.

[55] Shapiro S.S., Wilk M.B. (1965). An analysis of variance test for normality (complete samples). Biometrika.

[56] Smalheiser N.R., Smalheiser N.R. (2017). Chapter 12—Nonparametric Tests. Data Literacy.

[57] Lionello P., Abrantes F., Gacic M., Planton S., Trigo R., Ulbrich U. (2014). The climate of the Mediterranean region: Research progress and climate change impacts. Reg. Environ. Change.

[58] Marchina C., Natali C., Bianchini G. (2019). The Po River water isotopes during the drought condition of the year 2017. Water.

[59] Peña-Angulo D., Vicente-Serrano S.M., Domínguez-Castro F., Lorenzo-Lacruz J., Murphy C., Hannaford J., Allan R.P., Tramblay Y., Reig-Gracia F., El Kenawy A. (2022). The complex and spatially diverse patterns of hydrological droughts across Europe. Water Resour. Res..

[60] Rakovec O., Samaniego L., Hari V., Markonis Y., Moravec V., Thober S., Hanel M., Kumar R. (2022). The 2018–2020 multi-year drought sets a new benchmark in Europe. Earths Future.

[61] Pugliese E., Giuliani C., Bolognesi G., Filippini M., Barbero M., Ehdaie M., Gargini A. (2025). Tracing groundwater in a fractured arenitic aquifer by DNA-labelled, silica encapsulated nanoparticles in the Northern Apennines (Italy). Hydrog. J..

[62] Fuchs L., Stevens L.E., Fulé P.Z. (2019). Dendrochronological assessment of springs effects on ponderosa pine growth, Arizona, USA. For. Ecol. Manag..

